# Interaction between the Type III Effector VopO and GEF-H1 Activates the RhoA-ROCK Pathway

**DOI:** 10.1371/journal.ppat.1004694

**Published:** 2015-03-04

**Authors:** Hirotaka Hiyoshi, Ryu Okada, Shigeaki Matsuda, Kazuyoshi Gotoh, Yukihiro Akeda, Tetsuya Iida, Toshio Kodama

**Affiliations:** 1 Laboratory of Genomic Research on Pathogenic Bacteria, International Research Center for Infectious Diseases, Research Institute for Microbial Diseases, Osaka University, Suita, Osaka, Japan; 2 Laboratory of Clinical Research on Infectious Diseases, International Research Center for Infectious Diseases, Research Institute for Microbial Diseases, Osaka University, Suita, Osaka, Japan; 3 Microbe Repository Unit, International Research Center for Infectious Diseases, Research Institute for Microbial Diseases, Osaka University, Suita, Osaka, Japan; Purdue University, UNITED STATES

## Abstract

*Vibrio parahaemolyticus* is an important pathogen that causes food-borne gastroenteritis in humans. The type III secretion system encoded on chromosome 2 (T3SS2) plays a critical role in the enterotoxic activity of *V. parahaemolyticus*. Previous studies have demonstrated that T3SS2 induces actin stress fibers in various epithelial cell lines during infection. This stress fiber formation is strongly related to pathogenicity, but the mechanisms that underlie T3SS2-dependent actin stress fiber formation and the main effector have not been elucidated. In this study, we identified VopO as a critical T3SS2 effector protein that activates the RhoA-ROCK pathway, which is an essential pathway for the induction of the T3SS2-dependent stress fiber formation. We also determined that GEF-H1, a RhoA guanine nucleotide exchange factor (GEF), directly binds VopO and is necessary for T3SS2-dependent stress fiber formation. The GEF-H1-binding activity of VopO via an alpha helix region correlated well with its stress fiber-inducing capacity. Furthermore, we showed that VopO is involved in the T3SS2-dependent disruption of the epithelial barrier. Thus, VopO hijacks the RhoA-ROCK pathway in a different manner compared with previously reported bacterial toxins and effectors that modulate the Rho GTPase signaling pathway.

## Author Summary

Many bacterial pathogens manipulate the actin cytoskeleton of mammalian cells to establish pathogenesis via invasion, to evade killing by phagocytes, to disrupt a barrier function, and to induce inflammation caused by translocation type III secretion (T3S) effector proteins. We demonstrated that the T3S effector protein (VopO) of the enteric pathogen *Vibrio parahaemolyticus* induced robust actin stress fiber formation in infected host cells. Furthermore, this actin rearrangement induced barrier disruption in a colon epithelial cell line. Although many types of effector proteins have been reported, VopO does not share homology with previously reported effector proteins, and no putative functional motifs could be identified. Finally, we determined that the direct binding of VopO to a RhoA guanine nucleotide exchange factor (GEF) is a key step in the induction of stress fiber formation. These findings indicate that VopO plays a unique role in the pathogenicity of *V. parahaemolyticus*.

## Introduction

*Vibrio parahaemolyticus* is a Gram-negative halophilic bacterium that causes acute gastroenteritis in humans after the consumption of contaminated raw or undercooked seafood. The emergence of pandemic strains poses a worldwide health threat [1]. *V. parahaemolyticus* possesses two type III secretion systems (T3SSs): T3SS1 and T3SS2 [2]. A T3SS is a multisubunit molecular system that delivers bacterial proteins known as effectors directly to the plasma membrane or into the cytoplasm of infected host cells. The translocated effectors then modify certain functions of the host cell by disrupting normal cell signaling processes [3].

T3SS2, which is encoded on chromosome 2, is a major contributor to the enterotoxic effects observed in several animal models [4–7]. The T3SS2-related gene cluster is encoded in an 80-kb pathogenicity island (Vp-PAI), which is conserved exclusively in pathogenic strains [8,9]. Recently, we demonstrated that the F-actin binding T3SS2 effector VopV is necessary for enterotoxicity [10]. During the identification of VopV, we identified several candidate effector genes that are encoded in the Vp-PAI region, but their roles in the pathogenicity of *V. parahaemolyticus* remain unknown. Consequently, the precise pathogenic mechanisms underlying *V. parahaemolyticus* infections are not fully understood.

Many bacterial pathogens manipulate the actin cytoskeleton of the host cell using diverse mechanisms during infection [11]. Tissue culture analysis has shown that *V. parahaemolyticus* T3SS2 causes two dramatic changes in the actin cytoskeleton: the accumulation of F-actin beneath bacterial microcolonies and the induction of actin stress fibers [10,12]. At least three T3SS2 effectors, *i*.*e*., VopV, VopL, and VopC, have been identified as actin cytoskeleton modification effectors. VopV exhibits an F-actin binding activity and is responsible for the F-actin accumulation phenotype [10]. VopL contains three Wiskott-Aldrich syndrome protein homology 2 (WH2) motifs, and it elicits an Arp2/3-independent actin nucleation activity and the induction of actin stress fiber formation when expressed in host cells [12]. However, Liverman *et al*. reported that *vopL* deficiency only resulted in modest reductions in the amount of stress fibers formed during infection, thereby suggesting that effector(s) other than VopL may contribute to this activity during *V. parahaemolyticus* infection.

Recently, we identified VopC, which deamidates Rac1 and Cdc42, and it is homologous to a cytotoxic necrotizing factor of uropathogenic *Escherichia coli*. We found that this effector is involved in the T3SS2-dependent formation of actin stress fibers via the activation of Rac1 [7]. In the absence of VopC, *V. parahaemolyticus* induces the formation of long, branched, and curved F-actin filaments instead of actin stress fibers in Caco-2 cells. This cytoskeletal modification is completely dependent on T3SS2. In addition, the activation of Rac1 alone is not sufficient to induce stress fiber formation in the absence of bacterial infection. These observations suggest that the formation of complete stress fibers by *V. parahaemolyticus* requires the coordinated action of VopC with other T3SS2 effector(s).

In this study, we identified a novel actin cytoskeleton-manipulating T3SS2 effector called VopO. VopO induces a high level of stress fiber formation in the host cell by activating the RhoA-ROCK pathway. We also determined that VopO binds directly to GEF-H1, a RhoA guanine nucleotide exchange factor (GEF), and that the GEF-H1-binding activity of VopO is correlated with its stress fiber formation activity. In addition, VopO-dependent stress fiber formation disrupts the epithelial barrier *in vitro*, as observed previously *in vivo* in infected intestinal tissue [5,13]. A number of bacterial toxins and effectors that activate or inactivate small GTPases via the direct modification or mimicry of GEFs or GTPase-activating proteins (GAPs) have been identified [14,15], but this is the first report of an effector or a toxin that activates GEFs via direct binding. Overall, these results suggest that VopO is a novel effector with a different mode of action compared with previously reported effectors and toxins that modulate the Rho GTPase signaling pathway.

## Results

### The RhoA-ROCK pathway is essential for T3SS2-dependent stress fiber formation

Previous studies have revealed that two effectors, VopC and VopL, are involved in T3SS2-dependent actin stress fiber formation. Recently, we demonstrated that VopC deamidates and activates Rac1 in infected cells and promotes stress fiber assembly. However, in contrast to a T3SS2-deficient mutant, the *vopC* deletion mutant still induces the formation of long, branched, and curved F-actin filaments in Caco-2 cells [7]. VopL has been reported to contribute to F-actin stress fiber formation [12]. Therefore, we first investigated whether the induction of T3SS2-dependent stress fibers in HeLa and Caco-2 cells is completely dependent on VopL (S1A, B Fig.).

**Fig 1:**
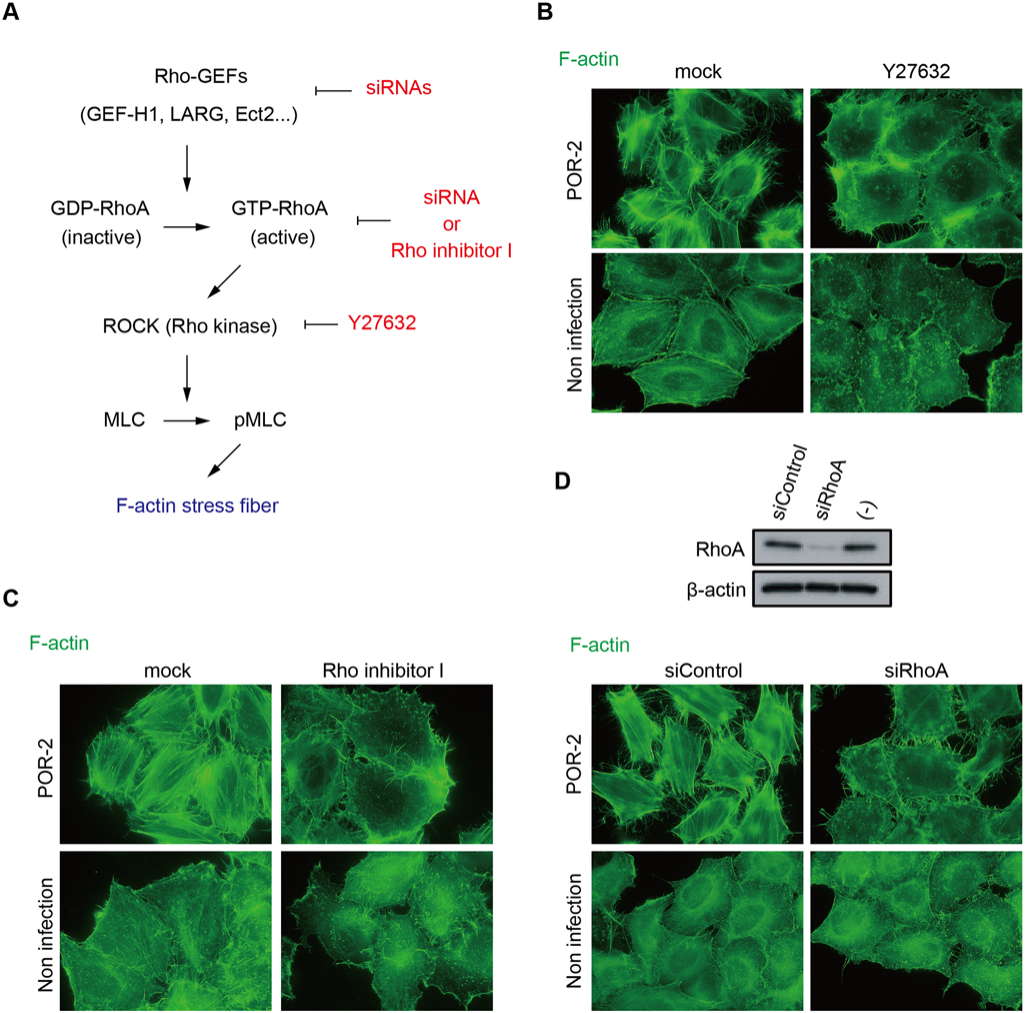
The RhoA-ROCK pathway is essential for T3SS2-dependent stress fiber formation. (A) Schematic model of stress fiber formation via the RhoA-ROCK pathway and the inhibitors/siRNAs used in this study HeLa cells were treated with 10 μμM ROCK inhibitor Y27632 for 1 h (B) or 2 μμg/mL RhoA inhibitor I for 2 h (C) prior to infection. Next, the cells were infected with POR-2 (a *tdhAS*- and T3SS1-deficient strain) for 3 h, fixed, and stained to detect F-actin with Alexa-488 phalloidin. (D) Silencing of RhoA by siRNA transfection. At 24 h after siRNA transfection, HeLa cells were infected with POR-2 for 3 h. The cells were then fixed and stained with Alexa-488 phalloidin. The silencing effect on RhoA protein expression was confirmed by western blot analysis. A scrambled target sequence siRNA was used as a negative control (siControl).

In agreement with the results of a previous study [12], in both cell types, we observed that the formation of actin stress fibers was somewhat attenuated after infection with a *vopL*-deficient strain (POR-2*∆vopL*) compared with the formation resulting from infection with the parent strain (POR-2). However, stress fibers were still observed in POR-2*∆vopL*-infected cells, whereas no fibers were observed after infection with a T3SS2-deficient strain (POR-2*∆vcrD2*) or in an uninfected control, suggesting that an unidentified effector is essential for stress fiber formation. The small GTPase RhoA and its downstream effector Rho-associated kinase are major mediators of stress fiber formation [16] (Fig. 1A). GTP binding and hydrolysis induce the conversion of RhoA between GTP-bound (active) and GDP-bound (inactive) forms [17]. The conversion of GTPases from an inactive to an active state is mediated by GEFs. Activated RhoA then propagates downstream signaling by binding to effector proteins such as ROCK. Activated ROCK leads to the formation of contractile bundles of F-actin via the phosphorylation of myosin light chain (MLC) [18,19]. Therefore, to determine whether the RhoA-ROCK pathway is required for T3SS2-dependent stress fiber formation, we employed a ROCK inhibitor (Y27632) and a Rho inhibitor (Rho inhibitor I, a permeable C3 exoenzyme from *Clostridium botulinum* that inhibits RhoA, RhoB, and RhoC in living cells). Treatment with either the ROCK inhibitor or the Rho inhibitor completely abolished the POR-2-induced formation of stress fibers (Figs. 1B and 1C).

We also examined the requirement for RhoA in stress fiber formation by silencing RhoA using siRNA (Fig. 1D). RhoA knockdown reduced the stress fiber formation induced by POR-2 infection (Fig. 1D), indicating that the RhoA-ROCK pathway is essential for T3SS2-dependent stress fiber formation. The T3SS2 effector VopC selectively deamidates and activates Rac1 and CDC42, but not RhoA, in infected cells both *in vitro* and *in vivo* [7]. VopL binds directly to actin and enhances actin filament assembly *in vitro* [12]. This activity of VopL does not require any Rho GTPases. Furthermore, there are no previous reports of T3SS2 effectors activating RhoA. Overall, these results strongly indicate the existence of an unidentified T3SS2 effector that activates the RhoA-ROCK pathway, thereby inducing stress fiber formation.

### Identification of the stress fiber-inducing T3SS2 effector, VopO

**Fig 2:**
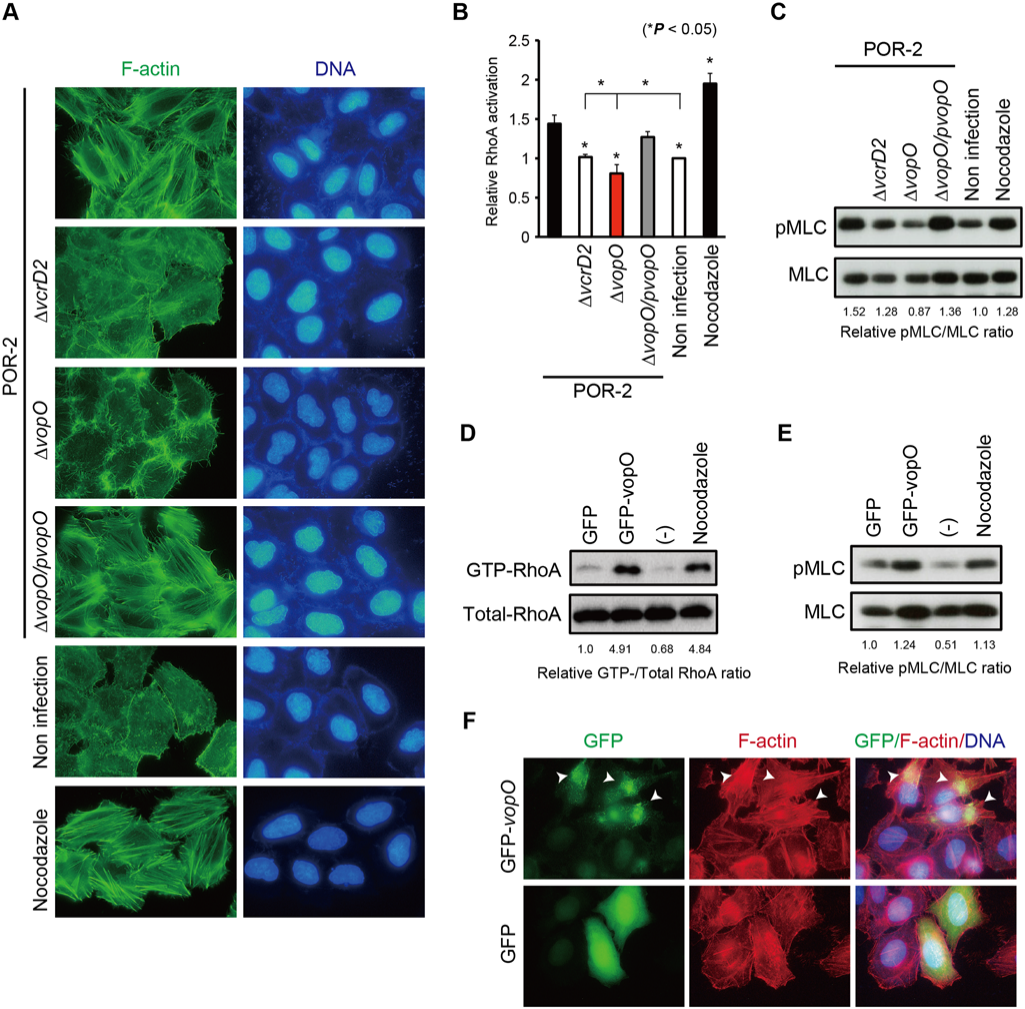
Identification of the stress fiber formation-inducing T3SS2 effector VopO. (A) HeLa cells were infected with POR-2, POR-2*∆vcrD2* (a T3SS1- and T3SS2-deficient strain), POR-2*∆vopO* (a *vopO* mutant strain derived from POR-2), or POR-2*∆vopO/pvopO* (a strain complemented with the *vopO* gene) for 3 h, or were treated with 10 mM nocodazole for 1 h. After infection or nocodazole treatment, the cells were stained to detect F-actin (green) and cellular and bacterial DNA (blue). (B) G-LISA was used to evaluate the relative RhoA activation level in cells infected with isogenic *V. parahaemolyticus* mutant strains for 150 min or treated with 10 mM nocodazole for 30 min. The asterisks indicate results that differ significantly from those obtained using the parent strain (POR-2) (*p < 0.05). The error bars indicate the standard errors for experiments performed in triplicate. (C) Phosphorylated myosin light chain (pMLC) levels were evaluated via western blot analysis of cells infected with isogenic *V. parahaemolyticus* mutant strains for 3 h or treated with 10 mM nocodazole for 1 h. The infected cell lysates were probed with anti-pMLC or anti-MLC antibodies. (D) The GTP-bound (active) RhoA levels were evaluated using a rhotekin pull-down assay in cells transfected with a GFP-fused VopO-expression construct (GFP-vopO) or empty GFP vector (GFP). The precipitates (GTP-RhoA) and total cell lysates (Total-RhoA) were probed with an anti-RhoA antibody. (E) The pMLC levels in cells transfected with a GFP-fused VopO-expression construct (GFP-vopO) or empty GFP vector (GFP) were evaluated by western blot analysis. (F) Visualization of GFP (green), F-actin (red), and cellular DNA (blue) in cells transfected with a VopO-expression construct (GFP-vopO) or empty GFP vector (GFP). The arrowheads indicate GFP-VopO-expressing cells.

Next, we aimed to identify the effector responsible for T3SS2-dependent stress fiber formation. After screening candidate ORFs encoded within the Vp-PAI region, a known pathogenicity island in pathogenic strains [20], we observed that deletion of the *vopO* gene (*vpa1329*: Gene ID 1192024) caused a dramatic change in actin stress fiber formation. Stress fibers were not detected when a *vopO*-deficient strain (POR-2*∆vopO*) was used to infect either HeLa cells (Fig. 2A) or Caco-2 cells (S1A Fig.). The lack of stress fiber induction when cells were infected with the POR-2*∆vopO* strain was rescued by *in trans* complementation with the *vopO* gene (POR-2*∆vopO/pvopO*). Immunoblotting analysis using anti-VopO antibodies revealed that VopO is specifically secreted into the culture medium via T3SS2 (S2A Fig.). Deletion of the *vopO* gene had no effect on the secretion of T3SS2 translocon proteins (VopB2 and VopD2) [21], the secretion of effector proteins involved in stress fiber formation (VopL and VopC) [7,12] (S2A Fig.), or T3SS2-dependent cytotoxicity against Caco-2 cells [22], which is a characteristic effect of T3SS2 *in vitro* (S2B Fig.).

The enterotoxic activity of the *vopO*-deficient strain (POR-2*∆vopO*) appeared to be reduced slightly compared with that of the POR-2 strain, and the enterotoxic activity of the complemented strain appeared to be slightly higher than that of the *vopO*-deficient strain; however, the differences between the enterotoxic effects of these strains were not significant (S2C Fig.). Interestingly, VopO was involved in the T3SS2-mediated cell invasion phenotype, which was recently identified as a phenotype with VopC activity (S2D Fig.) [7,23]. These observations indicate that VopO is not required for the full secretory function of T3SS2 (S1 Text), thereby suggesting that VopO is a T3SS2 effector involved in the induction of stress fiber formation and the invasive activity of *V. parahaemolyticus*.

We then examined whether VopO is involved in the activation of the RhoA-ROCK pathway. As a positive control in the subsequent assays, we used nocodazole, a microtubule destabilizer that induces stress fiber formation via the activation of RhoA [24]. As shown in Fig. 2B, RhoA activation by the parent strain (POR-2) was significantly higher than that induced by either its T3SS2-deficient derivative (POR-2*∆vcrD2*) or an uninfected control. However, the level of RhoA activation was significantly lower in cells infected with the *vopO*-deficient strain (*∆vopO*) than that in cells infected with the POR-2*∆vcrD2* strain or in uninfected control cells (Fig. 2B). This result suggests that balance of the Rho GTPase activation shifted from RhoA to Rac1 and Cdc42 because of activation via the other T3SS2 effector, VopC, which directly activates both Rac1 and Cdc42 [7,23]. Similar results were obtained by determining the amount of phosphorylated MLC (pMLC) (Fig. 2C). The amount of pMLC increased in a functional T3SS2-dependent manner. However, the amount of pMLC was lower in POR-2*∆vopO*-infected cells than that in cells infected with the POR-2*∆vcrD2* strain or uninfected control cells. These results suggest that VopO has an important role in the T3SS2-dependent activation of the RhoA-ROCK pathway in infected cells.

Next, we used a transfection assay to determine whether VopO itself activates the RhoA-ROCK pathway and subsequently induces stress fiber formation. Both GTP-bound RhoA (GTP-RhoA, the active form of RhoA, Fig. 2D) and pMLC (Fig. 2E) increased significantly when GFP-fused VopO was transiently expressed in HeLa cells. Moreover, the activation of RhoA and the augmentation of pMLC in GFP-VopO-expressing cells coincided with the formation of thick and massive actin fibers at the site of GFP-VopO protein localization (Fig. 2F, arrowheads). These findings indicate that VopO alone can activate the RhoA-ROCK pathway and induce stress fiber formation.

### GEF-H1, a VopO binding partner, is required for VopO-induced stress fiber formation

**Fig 3:**
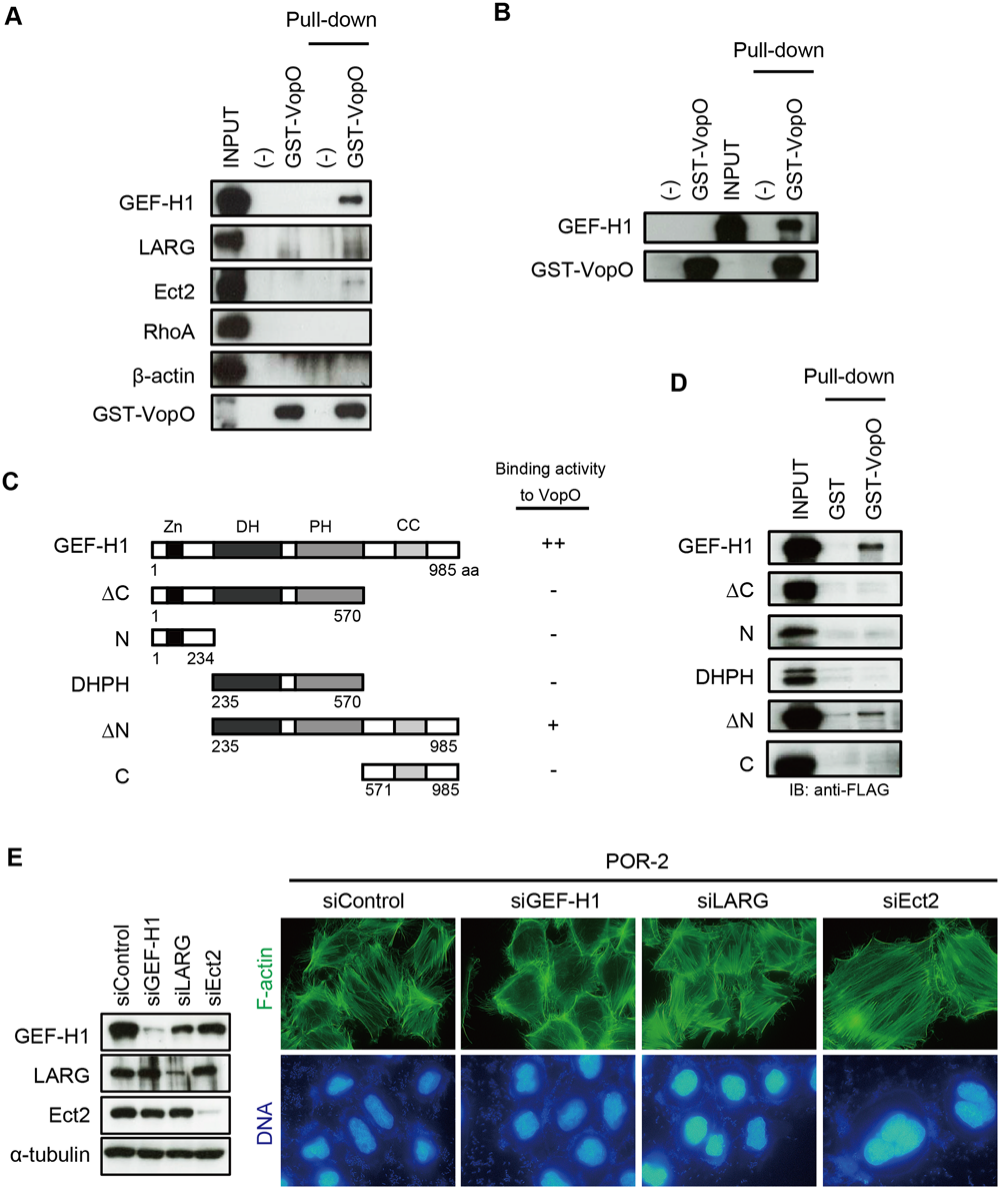
GEF-H1, a VopO binding partner, is necessary for VopO-induced stress fiber formation. (A) Identification of GEF-H1 as a VopO binding partner using a GST pull-down assay. Purified GST-VopO was mixed with a HeLa cell lysate, and the proteins retained on the glutathione beads were separated by SDS-PAGE. The proteins were detected with anti-GEF-H1, anti-LARG, anti-Ect2, anti-RhoA, and anti-β-actin antibodies. Lane 1, total HeLa cell lysate (INPUT); lane 2, eluate from glutathione beads alone; lane 3, eluate from GST-VopO bound to glutathione beads; lane 4, cell lysate associated with glutathione beads; and lane 5, cell lysate associated with GST-VopO bound to glutathione beads. (B) Direct binding of VopO to GEF-H1. Lane 1, glutathione beads alone; lane 2, purified GST-VopO; lane 3, recombinant 3×FLAG-tagged GEF-H1 (INPUT); lane 4, recombinant 3×FLAG-tagged GEF-H1 associated with glutathione beads; and lane 5, 3×FLAG-tagged GEF-H1 associated with GST-VopO bound to glutathione beads. Proteins were detected with anti-FLAG or anti-GST antibodies. (C) Diagram showing the recombinant truncated GEF-H1 proteins and summaries of their VopO-binding properties. (D) VopO-binding activity of truncated GEF-H1 proteins. After a pull-down assay using purified GST-VopO and recombinant 3×FLAG-tagged truncated GEF-H1 proteins, as shown in (C), GEF-H1 protein was detected using anti-FLAG antibody. (E) Effects of silencing GEF-H1, LARG, or Ect2 via siRNA on T3SS2-dependent stress fiber formation. At 24 h after the siRNA transfection, the cells were infected with POR-2 for 3 h. After infection, the cells were fixed and stained to detect F-actin (green) and DNA (blue). The silencing effects on the expression of each protein were confirmed by western blotting. A scrambled target sequence was used as a negative control (siControl).

We demonstrated the importance of the RhoA-ROCK pathway in VopO-dependent stress fiber induction. The transition of a small GTPase from an inactive to an active state is mediated by GEFs (Fig. 1A). Sixty-nine types of Rho GEFs have been reported previously [25], some of which contribute to the activation of RhoA [26,27]. We observed that T3SS2-dependent stress fiber formation was also blocked in dominant-negative RhoA-expressing cells (S3 Fig.). Therefore, we hypothesized that VopO may interact with at least one molecule located upstream of RhoA. A T3S effector EspG of enteropathogenic *Escherichia coli* (EPEC) induces stress fiber formation and is mediated by GEF-H1 activation [28]. GEF-H1 (Lfc in humans), which is a microtubule-regulated Rho GEF, plays a dominant role in RhoA activation [29]. This information let us to hypothesize that VopO might associate with GEF-H1 to induce the stress fiber. To test this hypothesis, we performed a pull-down assay using purified glutathione S-transferase (GST)-fused VopO (GST-VopO) and a HeLa cell lysate. We observed that GEF-H1 coprecipitated with GST-VopO. By contrast, two other RhoA GEFs, LARG and Ect2 [26], did not interact strongly with VopO (Fig. 3A). Interactions were not detected between VopO and RhoA or β-actin. Thus, we confirmed the direct binding of VopO with the recombinant full-length GEF-H1 protein, which was prepared using an *in vitro* translation system.

As shown in Fig. 3B, the full-length GEF-H1 protein coprecipitated with GST-VopO. GEF-H1 contains four domains: a zinc-finger motif-containing region (Zn), dbl-homology (DH), pleckstrin homology (PH), and α-helical coiled-coil (CC) domains (Fig. 3C) [29]. The DH and PH domains are conserved in the Rho GEF family and are required for its GEF activity [25]. By contrast, the Zn and CC domains are unique to GEF-H1 and they are involved in the regulation of GEF-H1 activity [29]. Thus, we used a pull-down assay to identify GEF-H1 domains that contribute to the binding activity to VopO (Fig. 3D). An N-terminally truncated GEF-H1 (∆N) protein retained relatively weak binding activity with VopO, whereas all of the truncated proteins lost their ability to bind to VopO. Because we could not identify a specific VopO-binding domain in GEF-H1, we hypothesize that VopO might specifically recognize the higher-order structure of GEF-H1. We then assessed the requirement for GEF-H1 in *V. parahaemolyticus-*induced stress fiber formation using siRNA. As shown in Fig. 3E, no changes in stress fibers were observed when the RhoA GEFs LARG and Ect2 were silenced. By contrast, cells in which only GEF-H1 was silenced exhibited diminished stress fiber formation. Overall, these findings suggest that VopO may target GEF-H1 to induce stress fiber formation. We explored this possibility in the following experiments.

### A predicted α-Helix region (H2) in VopO is required for GEF-H1 binding and stress fiber formation

**Fig 4:**
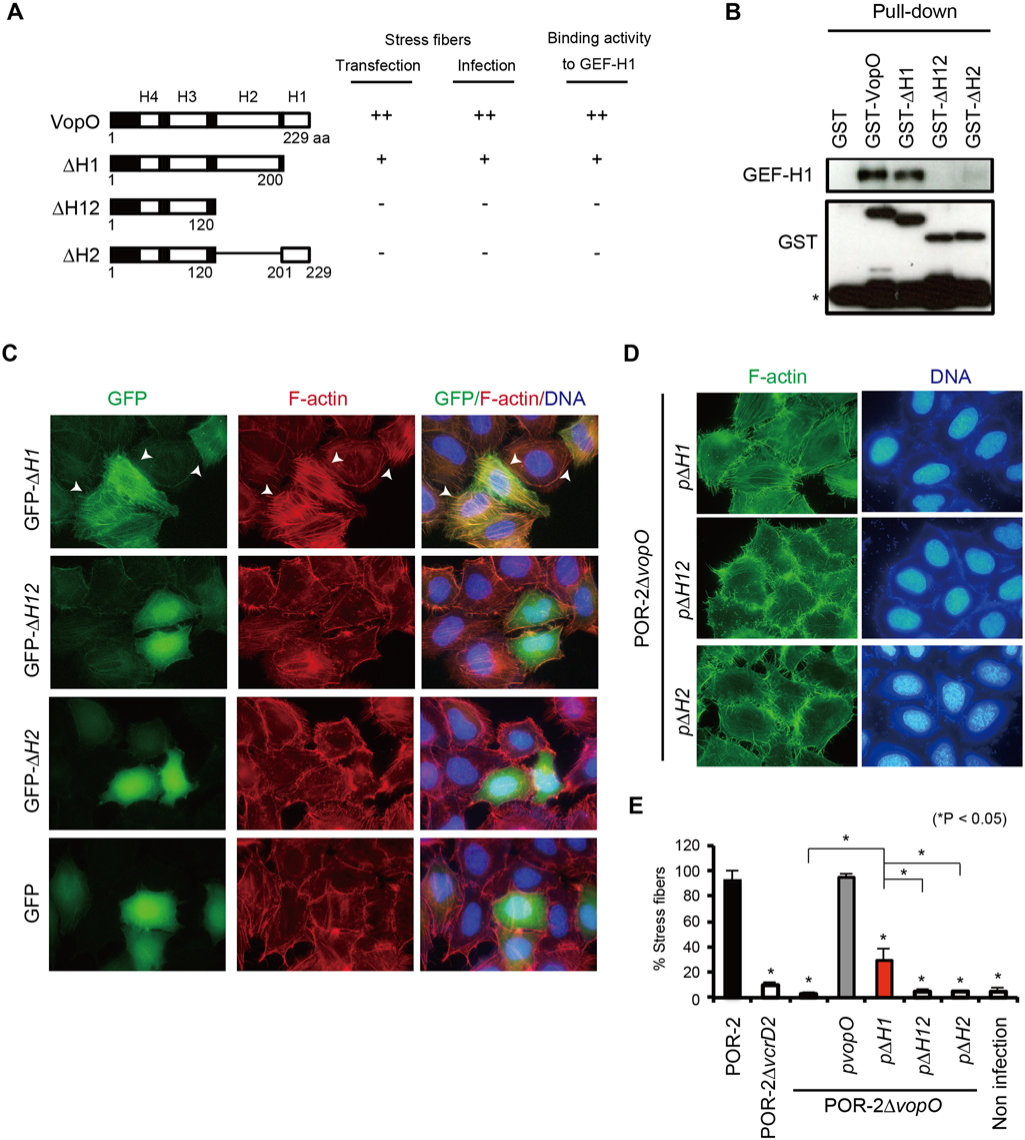
A predicted α-helix region in VopO (H2) is required for both GEF-H1 binding and stress fiber formation. (A) Diagram showing the recombinant truncated VopO proteins and summaries of their stress fiber-inducing activity, as determined by transfection and infection assays, and their GEF-H1-binding properties. (B) Identification of the GEF-H1-binding domain of VopO. The GEF-H1-binding activity of truncated VopO was evaluated using a GST pull-down assay. The 3xFLAG GEF-H1 and truncated GST-VopO proteins were detected using anti-FLAG or anti-GST antibodies, respectively. The asterisk indicates GST or a breakdown product. (C) Stress fiber-inducing activity of truncated VopO proteins in transfected cells. HeLa cells were transfected with GFP-fused truncated VopO constructs. The cells were stained to detect F-actin (red) and cellular DNA (blue). (D) HeLa cells were infected with *vopO* deletion mutants that expressed truncated VopO proteins and the stress fiber-inducing activity was evaluated by staining to detect F-actin (green) and cellular DNA (blue). (E) Percentage of cells exhibiting actin stress fibers after infection with *vopO* deletion mutants expressing truncated VopO proteins. One hundred cells from several fields were analyzed by microscopy in each experiment to determine whether stress fiber formation was induced. The means ± standard errors are presented for experiments conducted in triplicate. The asterisks indicate statistically significant differences (*p < 0.05).

VopO does not share any motifs or any sequence homology with known proteins; however, the Chou-Fasman secondary structure prediction program (http://cib.cf.ocha.ac.jp/bitool/MIX/) revealed that VopO possesses at least four α-helix regions: H1, H2, H3, and H4 (S4 Fig.). We used several truncated forms of VopO (Fig. 4A) to identify the α-helix region(s) in VopO responsible for binding to GEF-H1. In VopO where the first C-terminal α-helix region (∆H1) was truncated, the GEF-H1-binding activity was attenuated slightly compared with that of the full-length VopO (Fig. 4B). By contrast, in the VopO proteins that lacked the second C-terminal α-helix region (H2), ∆H12 or ∆H2, the GEF-H1-binding activity was reduced dramatically.

Next, we used cell transfection to evaluate the stress fiber formation activity of these truncated VopO proteins (Fig. 4C). The stress fibers of ∆H1-expressing cells were somewhat weaker than those of the full-length VopO-expressing cells (Figs. 2F and 4C). By contrast, ∆H12 and ∆H2 did not induce any stress fiber formation. In *V. parahaemolyticus*-infected cells, the stress fiber-inducing activity of the POR-2*∆vopO* strain was restored completely or partially by complementation with full-length or ∆H1 VopO, respectively (Figs. 2A, 4D and 4E). By contrast, no stress fiber induction activity was observed in cells infected with the ∆H12- or ∆H2-complemented strains (Figs. 4D and 4E). As summarized in Fig. 4A, the stress fiber-inducing activity of VopO was strongly correlated with its GEF-H1-binding activity.

### VopO does not disrupt the microtubule network

The interaction between GEF-H1 and microtubules is important for GEF-H1 inactivation [30]. Several T3SS effectors, such as EspG, EspG2, and Orf3 from EPEC, release and activate GEF-H1 by disrupting the host microtubule network following stress fiber induction in infected host cells [28,31]. Therefore, we explored the possibility that VopO might disrupt the microtubule structure. The transient expression of DsRed-fused VopO in cells induced stress fibers (S5A Fig.) but did not induce the destruction of the microtubule network that is observed during transient EspG expression in host cells (S5B Fig.) [32]. In addition, several DsRed-VopO puncta appeared to colocalize with GFP-fused GEF-H1 but they did not significantly affect the association between GEF-H1 and microtubules (S5C Fig.). Furthermore, although microtubule disruption is reported to be EspG-dependent in cells infected with EPEC [28], VopO-dependent microtubule disruption was not observed in *V. parahaemolyticus*-infected cells (S5D Fig.). Taken together, these results indicate that GEF-H1 is a primary target of VopO during the induction of stress fiber formation. However, the mechanism that allows VopO to activate GEF-H1 is different from that reported for microtubule-destabilizing T3S effectors.

### VopO disrupts the epithelial barrier

The intestinal epithelial barrier, which includes tight junctions, plays an important role in defense against the invasion of pathogens and commensal microbiota into the lamina propria [33–35]. Junctional adhesion molecule-A-deficient mice, which have a highly permeable intestinal epithelial barrier, are susceptible to enterocolitis [34]. Several studies have also reported intimate relationships between stress fiber formation and the homeostasis of the intestinal epithelial barrier [36–38]. Therefore, we investigated whether stress fiber formation induced by VopO affects the integrity of the epithelial barrier.

**Fig 5:**
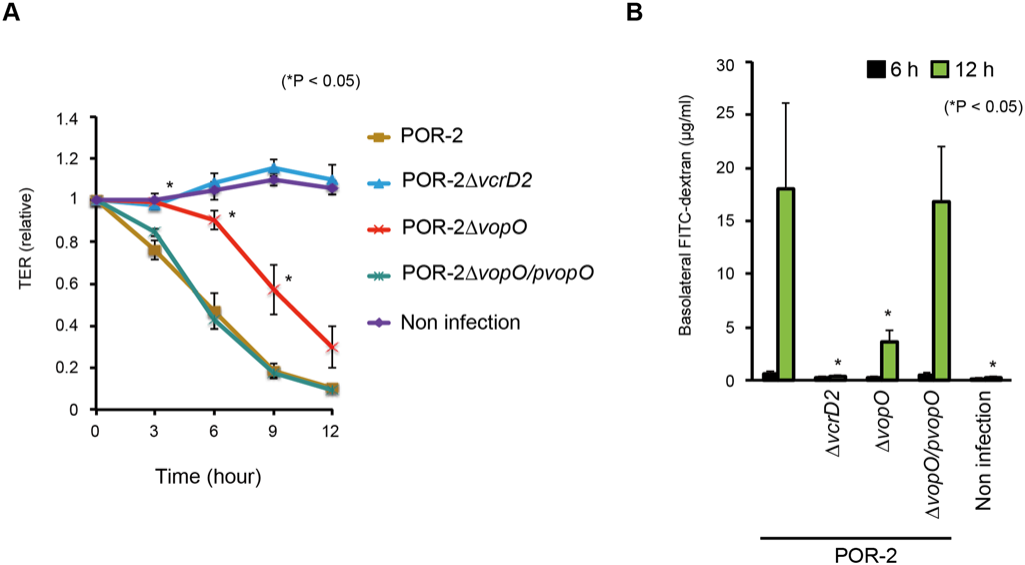
VopO disrupts the epithelial barrier. Polarized Caco-2 cells in Transwell plates were infected with isogenic mutant strains of *V. parahaemolyticus* for the indicated time, and their barrier functions were evaluated using trans-epithelial resistance (TER) measurements (A) and the FITC-dextran leakage test (B). The asterisks indicate results that differed significantly from those obtained using the parent (POR-2) strain (*p < 0.05). The error bars indicate the standard errors for experiments performed in triplicate.

The integrity of the epithelial barrier was evaluated by measuring the trans-epithelial resistance (TER) of polarized Caco-2 cells (Fig. 5A). The TER value of Caco-2 cells infected with the parent strain (POR-2) decreased over time. By contrast, the TER value of cells infected with a T3SS2-deficient strain (POR-2*∆vcrD2)* was nearly identical to that of uninfected control cells. The TER value of cells infected with a *vopO*-deficient strain (POR-2*∆vopO*) declined significantly more slowly than that of POR-2-infected cells. We also confirmed the VopO-dependent disruption of the epithelial barrier in a FITC-dextran leakage assay [39]. A mixture of POR-2 and FITC-dextran was used to challenge the apical side of polarized Caco-2 cells and its leakage into the basolateral side was monitored. The amount of basolateral dextran increased dramatically at 12 h after the infection of cells with POR-2 compared with POR-2*∆vcrD2*-infected cells or uninfected control cells (Fig. 5B). The basolateral dextran levels were significantly lower in POR-2*∆vopO*-infected cells than those in cells infected with POR-2. Moreover, the reductions in both the TER value and the amount of basolateral dextran observed for the POR-2*∆vopO* strain were rescued by *in trans* complementation with the *vopO* gene (Figs. 5A and 5B).

The comparable cytotoxicities of POR-2*∆vopO* and POR-2 against Caco-2 cells (S2B Fig.), suggest that disruption of the VopO-dependent epithelial barrier activity was not attributable to cytotoxicity. These results suggest that the stress fiber-inducing activity of VopO disrupts the epithelial barrier function.

## Discussion

T3SS2, which is encoded in Vp-PAI on chromosome 2 of *V. parahaemolyticus*, is essential for enterotoxicity in several animal models, thereby indicating that it is involved in the pathogenicity of this bacterium [4,5]. Recently, we identified VopV as an enterotoxic T3SS2 effector in a rabbit loop assay [10]. However, the Vp-PAI region encodes many hypothetical ORFs whose biological activities and roles in virulence are not fully understood. In the present study, we determined that a functionally undetermined ORF encoded by Vp-PAI, VPA1329 (VopO), is a novel T3SS2 effector of *V. parahaemolyticus*, which participates in disrupting the host epithelial barrier by inducing stress fiber formation.

During infection, bacterial pathogens use diverse mechanisms to manipulate the actin cytoskeleton of host cells [11]. In *V. parahaemolyticus*, T3SS2 has been reported to induce the accumulation of F-actin beneath bacterial microcolonies and the formation of actin stress fibers within infected host cells [10,12]. In this and previous studies, we have demonstrated that at least three T3SS2 effectors, VopO, VopL, and VopC, are involved in T3SS2-dependent stress fiber formation [7,10,12,23]. Although VopL induces stress fiber formation in transfected cells, it is not essential for this process (S1A, B Fig.) [12]. Furthermore, the actin filament assembly-enhancing activity of VopL, dose not require any Rho GTPases. A *vopC-*deficient strain caused the formation of long, branched, and curved F-actin filaments with a reticular appearance; these fibers were not observed in cells infected with a T3SS2-deficient strain (S1A Fig.) [7]. The expression of a constitutively active form of Rac1 restored the strain’s capacity to induce normal stress fiber formation. VopO activation of the RhoA-ROCK pathway via GEF-H1 binding is essential for stress fiber formation.

These observations indicate that activation of the RhoA-ROCK pathway by VopO is a requisite first step in the induction of stress fiber formation. The activity of VopL, which nucleates actin filaments, appears to enhance the efficiency of stress fiber formation. Stress fibers are contractile acto-myosin structures, which are attached to focal adhesions at both ends of the fiber. The focal complexes formed in lamellipodia are triggered by Rac1 activation [40]. Rac1-null primary mouse embryonic fibroblasts cannot form focal adhesion complexes or induce RhoA-regulated actin stress fiber formation [41]. A *vopC* deletion mutant lacks the ability to induce vinculin foci [7]. The focal complexes formed by VopC-activated Rac1 may be converted into focal adhesions, and this signaling cascade is necessary for the formation of robust actin stress fibers.

Stress fiber formation plays a role in the maintenance of the epithelial barrier against both pathogen invasion and commensal microbiota [24,33,34,36,38]. Inappropriate induction of stress fibers disrupts tight junctions and leads to several diseases [42]. A recent *in vivo* study demonstrated that *V. parahaemolyticus* disrupts the tight junction complex of small intestinal epithelial cells prior to inducing diarrhea in an infant rabbit oral infection model [5]. The involvement of VopO in this phenomenon was difficult to determine using a rabbit ileal loop test *in vivo*; however, based on TER measurements and FITC-dextran leakage assays, we detected VopO-dependent disruption of the epithelial barrier (Figs. 5A and 5B). These results suggest that VopO is closely involved in the disruption of tight junction complexes, which is consistent with observations in the infant rabbit oral infection model.

*V. parahaemolyticus* is usually considered to be a noninvasive bacterial pathogen, but several recent studies have demonstrated that this bacterium can invade epithelial cells, which depends on VopC before replicating in the cytosol of the host cells [7,23,43]. In the present study, we found that a *vopO* deficient strain also abolished T3SS2-dependent invasive phenotype (S2D Fig.). Consequently, we hypothesize that a fairly complex mechanism is required for T3SS2-dependent invasion because the invasive capacity of this bacterium is needed to activate Cdc42, but not Rac1 and RhoA [7], and treatment with nocodazole, which disrupts microtubules and activates GEF-H1, inhibited the invasion of *V. parahaemolyticus* into HeLa and Caco-2 cells [23,44]. These observations indicate that, unlike VopO-dependent stress fiber formation, activation of the GEF-H1-mediated RhoA-ROCK pathway is not related to *V. parahaemolyticus* cell invasion. Further studies are needed to understand how VopO promotes invasion by *V. parahaemolyticus*.

T3S effectors activate Rho family small GTPases in diverse ways. Some effectors share the common motif Trp-xxx-Glu (WxxxE) and functionally mimic GEFs [15,45], whereas other effectors belong to the deamidating toxin family, the members of which directly modify (via deamidation/transglutamination) Rho family small GTPases [11]. In addition to these effectors, EspG, EspG2, and Orf3 in EPEC disrupt the microtubule network, thereby resulting in the release and activation of GEF-H1 [28,31]. However, VopO does not possess a WxxxE motif (or any type of functional motif), shares no sequence homology with these effectors, and does not disrupt microtubule structures in transfected or infected cells (S5B, C, and D Fig.) [32]. In addition, the deamidation of RhoA was not observed in cells infected with *V. parahaemolyticus* [7]. These results suggest that VopO hijacks the RhoA-ROCK pathway in a different manner compared with previously reported effectors and toxins.

In the present study, we clearly demonstrated that VopO binds directly to GEF-H1 and that the stress fiber-inducing activity of VopO was correlated with its GEF-H1 binding activity (Fig. 4A). GEF-H1 is a RhoA GEF, which is regulated by microtubule binding, phosphorylation, and protein-protein interactions [30,46–51]. The interaction between GEF-H1 and microtubules is particularly important for the suppression of GEF-H1 activation, where the unique N- and C-termini of GEF-H1 are responsible for its association with microtubules [30]. In a previous study, transient expression of either N- or C-terminal-truncated GEF-H1 resulted in higher GEF activity compared with the expression of full-length GEF-H1, thereby suggesting that these domains negatively regulate GEF activity via the DH and PH domains, which are required for the GEF activity of GEF-H1 [30]. Importantly, VopO binds to GEF-H1, which contains the C-terminal domain (Figs. 3C and D), but the expression of DsRed-fused VopO did not induce obvious alterations in GFP-GEF-H1 localization according to microscopic analysis (S5C Fig.). In addition, we did not observe any VopO-mediated increase in the activity of GEF-H1 with RhoA according to an *in vitro* Rho GEF exchange assay using mant-GTP. Therefore, the biochemical mechanism that allows VopO to target GEF-H1 and how it coordinates with other *V. parahaemolyticus* T3SS2 effectors during bacterial infection remain unclear. Further research is required to determine how VopO activates the RhoA-ROCK pathway via GEF-H1. A more detailed understanding of the functional mechanism of VopO may provide new insights into how pathogenic bacteria exploit the signaling pathways of Rho family small GTPases.

## Materials and Methods

### Bacterial strains and plasmids

*V. parahaemolyticus* strain RIMD2210633 (KP-positive, serotype O3:K6)[2] was obtained from the Pathogenic Microbes Repository Unit, International Research Center for Infectious Diseases, Research Institute for Microbial Diseases (Osaka University, Osaka, Japan). A four-primer polymerase chain reaction (PCR) technique was used to engineer an in-frame deletion mutation, as described previously [4]. All of the bacterial strains and plasmids used in this study are listed in the Supporting Information, S1 Table.

### RhoA activation

The activation of RhoA was estimated using a G-LISA RhoA Activation Assay Biochem Kit (Cytoskeleton Inc., Denver, CO, USA) or an EZ-Detect Rho Activation Kit (Thermo Fisher Scientific Inc., Waltham, MA, USA), according to the manufacturer’s instructions. One day prior to infection, the culture medium was exchanged with DMEM containing 0.25% fetal bovine serum. HeLa cells were infected with isogenic *V. parahaemolyticus* strains at a multiplicity of infection (MOI) of 10 for 150 min or treated with 10 μM nocodazole (Sigma-Aldrich, St. Louis, MO, USA) for 30 min as a positive control. After infection or nocodazole treatment, RhoA activation was evaluated by an ELISA (the G-LISA RhoA Activation Assay Biochem Kit, Cytoskeleton Inc., Denver, CO, USA). The amount of GTP-RhoA in the transfected HeLa cells was estimated via a rhotekin pull-down assay using an EZ-Detect Rho Activation Kit. The intensity of GTP-RhoA bands was measured using ImageJ software (NIH, Bethesda, MD, USA).

### Phosphorylation of MLC

HeLa cells were infected with *V. parahaemolyticus* strains at a MOI of 10 for 3 h or treated with 10 μM nocodazole for 30 min. The cell lysates were probed with p(Thr18/Ser19)-MLC and MLC antibodies (Cell Signaling Technology, Inc., Danvers, MA, USA).

### Immunofluorescence assay

HeLa or Caco-2 cells were infected with *V. parahaemolyticus* strains at a MOI of 10 for 3 h or treated with 10 μM nocodazole for 1 h. To inhibit stress fiber formation, 10 μM Y27632 (Sigma-Aldrich, St. Louis, MO, USA) or 2 μg/mL Rho inhibitor I (Cytoskeleton Inc., Denver, CO, USA) was added for 1 or 2 h prior to infection, respectively. After infection, the cells were washed with ice-cold phosphate-buffered saline (PBS) and fixed with 4% paraformaldehyde in PBS. The fixed cells were then stained for F-actin and DNA using Alexa-488 or rhodamine-conjugated phalloidin (Invitrogen, Carlsbad, CA, USA) and Hoechst 33258 (Sigma-Aldrich, St. Louis, MO, USA), respectively. Images were captured using a fluorescence microscope (Biozero BZ-8100, Keyence, Osaka, Japan) or a confocal laser microscope (FLUOVEW FV10i, Olympus, Tokyo, Japan).

### Transfection assay

The transfection of GFP fused with full-length or truncated VopO expression vectors was performed using Lipofectamine LTX reagent (Invitrogen, Carlsbad, CA, USA) according to the manufacturer’s instructions. At 15 h after transfection, the HeLa cells were washed with ice-cold PBS and fixed with 4% paraformaldehyde in PBS. The fixed cells were then stained for F-actin and nuclei using rhodamine phalloidin (Invitrogen, Carlsbad, CA, USA) and Hoechst 33258 (Sigma-Aldrich, St. Louis, MO, USA), respectively. Images were captured using a fluorescence microscope (Biozero BZ-8100, Keyence, Osaka, Japan).

### Pull-down assay

Confluent HeLa cells were washed with ice-cold PBS and lysed in 1 mL of ice-cold RIPA buffer [50 mM Tris-HCl (pH 8.0), 150 mM NaCl, 0.1% sodium dodecyl sulfate (SDS), 0.5% deoxycholic acid (DOC), and 1% NP-40] containing an EDTA-free protease inhibitor cocktail (Sigma-Aldrich, St. Louis, MO, USA). After agitation for 15 min at 4°C, the lysates were harvested with a cell scraper and centrifuged for 15 min at 10,000 × *g*. Recombinant 3xFLAG-tagged wild-type or truncated GEF-H1 proteins were prepared using TnT Quick Coupled Transcription/Translation Systems (Promega, Fitchburg, WI, USA), according to the manufacturer’s instructions. The lysates from the HeLa cells or recombinant GEF-H1 proteins were incubated with GST-VopO proteins and glutathione beads (GE Healthcare, Little Chalfont, UK) at 4°C for 4 h. The beads were washed with RIPA buffer and eluted with SDS sample buffer, and the eluates were separated by SDS-polyacrylamide gel electrophoresis (SDS-PAGE).

### Western blotting

The samples used for western blotting were separated by SDS-PAGE. After electrotransfer, the polyvinylidene fluoride (PVDF) membranes (Merck Millipore, Darmstadt, Germany) were probed with anti-GEF-H1, p(Thr18/Ser19)-MLC, MLC, anti-GST (Cell Signaling Technology, Inc., Danvers, MA, USA), anti-LARG, anti-Ect2, anti-RhoA, anti-β-actin, anti-α-tubulin, or anti-FLAG (Sigma-Aldrich, St. Louis, MO, USA) antibodies, followed by horseradish-peroxidase-conjugated goat anti-rabbit or rabbit anti-mouse antibodies (Zymed Laboratories, Inc., South San Francisco, CA, USA). The blots were developed using an ECL Western Blotting Kit (GE Healthcare, Little Chalfont, UK).

### TER measurement and FITC-dextran leakage assays

A total of 2 x 10^5^ Caco-2 cells were plated into Transwell chambers (Corning Inc., Corning, NY, USA) with a pore size of 0.4 μm (6.5 mm diameter) and cultured for 12–14 days. The medium was changed every 2 days until a steady-state TER (450–550 Ωcm^2^) was achieved. The cells were infected with *V. parahaemolyticus* strains, and the TER was measured using an epithelial voltmeter (EVOM, WPI Inc., Sarasota, FL, USA) at the indicated time. The FITC-dextran leakage assay was conducted as described previously [39]. Mixtures of bacteria and 4-kDa FITC-dextran (Sigma-Aldrich, St. Louis, MO, USA) were used to challenge the apical side of the Transwell chambers. At 6 and 12 h after infection, the amount of FITC-dextran on the basolateral side was measured using a PowerScan HT fluorescence plate reader (DS Pharma Biomedical Co., Ltd, Osaka, Japan).

### Silencing of RhoA and Rho GEFs by siRNA

Specific siRNAs for RhoA, GEF-H1, LARG, or Ect2 were purchased from Applied Biosystems. Silencer Select Negative Control #1 siRNA (Applied Biosystems, Foster City, CA, USA) was used as a negative control. The siRNAs were transfected using siPORT NeoFX Transfection Agent (Applied Biosystems, Foster City, CA, USA). At 24 h after transfection, the cells were subjected to western blot analysis to confirm the knockdown efficiency and used subsequently in the infection studies.

### Statistical analysis

All of the data are expressed as the mean and standard error based on at least three determinations per experimental condition. Student’s *t* tests that assumed unequal variances were used for the statistical analyses. *P* < 0.05 was considered significant.

## Supporting Information

S1 Fig Deletion of the *vopL* gene partially attenuates the T3SS2-dependent formation of actin stress fibers. Caco-2 cells (A) or HeLa cells (B) were infected with isogenic *V. parahaemolyticus* mutant strains at a MOI of 10 for 3 h. Then, the cells were fixed and stained to detect F-actin (green) and cellular and bacterial DNA (blue). Bars = 20 μm.
(TIF)

S2 Fig VopO is not required for the full secretory function of T3SS2. (A) Secreted protein profiles of POR-1 (*tdhAS*-deficient strain), POR-2 (T3SS1-deficient strain derived from POR-1), POR-3 (T3SS2-deficient strain derived from POR-1), POR-2*∆vcrD2* (T3SS1- and T3SS2-deficient strain derived from POR-1), and POR-2*∆vopO* (*vopO*-deficient strain derived from POR-2). The membranes were probed with anti-VopO, anti-VopB1 (translocon of T3SS1), anti-VopB2 (translocon of T3SS2), anti-VopD2 (translocon of T3SS2), anti-VopL (T3SS2 effector), or anti-VopC (T3SS2 effector) antibodies. (B) Effect of *vopO* deletion on T3SS2-dependent cytotoxicity against Caco-2 cells. Caco-2 cells were infected with isogenic mutants of POR-2 at a MOI of 10. At 6 h after infection, cytotoxic activity was evaluated by determining the amount of lactate dehydrogenase released. The asterisks indicate results that differ significantly from those obtained using the wild-type strain (*p < 0.01). The error bars indicate the standard errors for experiments performed in triplicate. n.s. = not significant. (C) Effect of *vopO* deletion on T3SS2-dependent enterotoxicity. The enterotoxicity of various isogenic *V. parahaemolyticus* mutant strains was evaluated using the rabbit ileal loop test. The fluid accumulation (FA) ratio in each loop was measured 18 h after infection. FA is the amount of accumulated fluid (in ml) per length (in cm) of ligated rabbit small intestine. The asterisks indicate results that differ significantly from those obtained using the wild-type strain (*p < 0.01). The error bars represent the standard errors. n.s. = not significant. (D) Effect of *vopO* deletion on T3SS2-mediated cell invasion. HeLa cells were infected with isogenic mutant strains of POR-2 at an MOI of 2 for 2 h. Frequencies of invasion are expressed relative to the invasion of POR-2, which was set at 1.0. The error bars indicate the standard errors for experiments performed in triplicate. The asterisks indicate results that differ significantly from those obtained using the POR-2 strain (*p < 0.01).
(TIF)

S3 Fig Stress fiber induction by *V. parahaemolyticus* is inhibited by the expression of dominant-negative (DN) RhoA. FLAG-tagged DN-RhoA (pMEPyori-RhoN19) or mock vector (pMEPyori) was transfected into HeLa cells. At 24 h after transfection, the cells were infected with a POR-2 strain at a MOI of 10 for 3 h. The cells were stained to detect transiently expressed DN-RhoA (red), F-actin (green), and cellular and bacterial DNA (blue).
(TIF)

S4 Fig Secondary protein structure of the VopO protein. The secondary structure of the VopO protein was predicted using the Chou-Fasman secondary structure prediction program (http://cib.cf.ocha.ac.jp/bitool/MIX/).
(TIF)

S5 Fig VopO does not disrupt the microtubule network. **(A)** Visualization of F-actin (green), DsRed (red), and cellular DNA (blue) in HeLa cells transfected with a VopO expression construct (DsRed-*vopO*) or empty DsRed vector (DsRed). (B) Effect of cellular VopO expression on the microtubule network. Cells transfected with a VopO-expression construct (DsRed-*vopO*) or empty DsRed vector (DsRed) were stained to detect tubulin (green) and cellular and bacterial DNA (blue). (C) Effect of cellular VopO expression on association of GEF-H1 with microtubules. A VopO expression construct (DsRed-*vopO*) or empty DsRed vector (DsRed) were co-transfected with GFP-GEF-H1 expressing vector (green). After staining to detect tubulin (cyan), the cells were examined by confocal microscopy. (D) Effect of *V. parahaemolyticus* infection on microtubule dynamics. HeLa cells were infected with a *vopO* mutant (POR-2*∆vopO*) and its complemented strain (POR-2*∆vopO/pvopO*) for 3 h. The cells were stained to detect tubulin (green), cellular and bacterial DNA (blue), and F-actin (red).
(TIF)

S1 Table Bacterial strains and plasmids used in this study. (DOCX)

S1 Text Supporting information. (DOCX)
