# Supplementary figures and images for: Interaction between the Type III Effector VopO and GEF-H1 Activates the RhoA-ROCK Pathway
Source: PLoS Pathog. 2015 Mar 4;11(3):e1004694. doi: 10.1371/journal.ppat.1004694 (PMC4349864; doi:10.1371/journal.ppat.1004694)

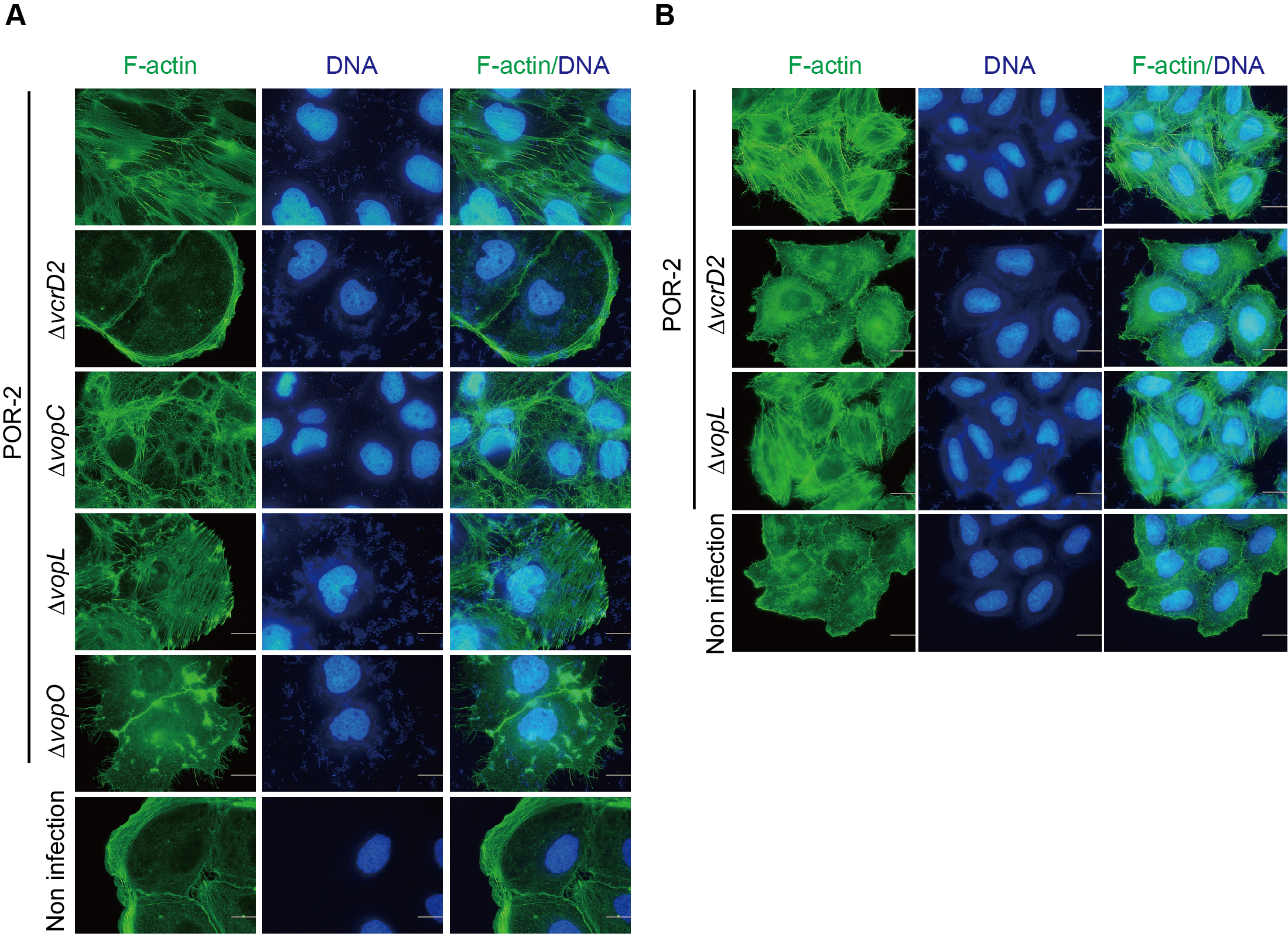

Supplement: S1 Fig — Caco-2 cells (A) or HeLa cells (B) were infected with isogenic V. parahaemolyticus mutant strains at a MOI of 10 for 3 h. Then, the cells were fixed and stained to detect F-actin (green) and cellular and bacterial DNA (blue). Bars = 20 μm. (TIF) [file ppat.1004694.s001.tif]

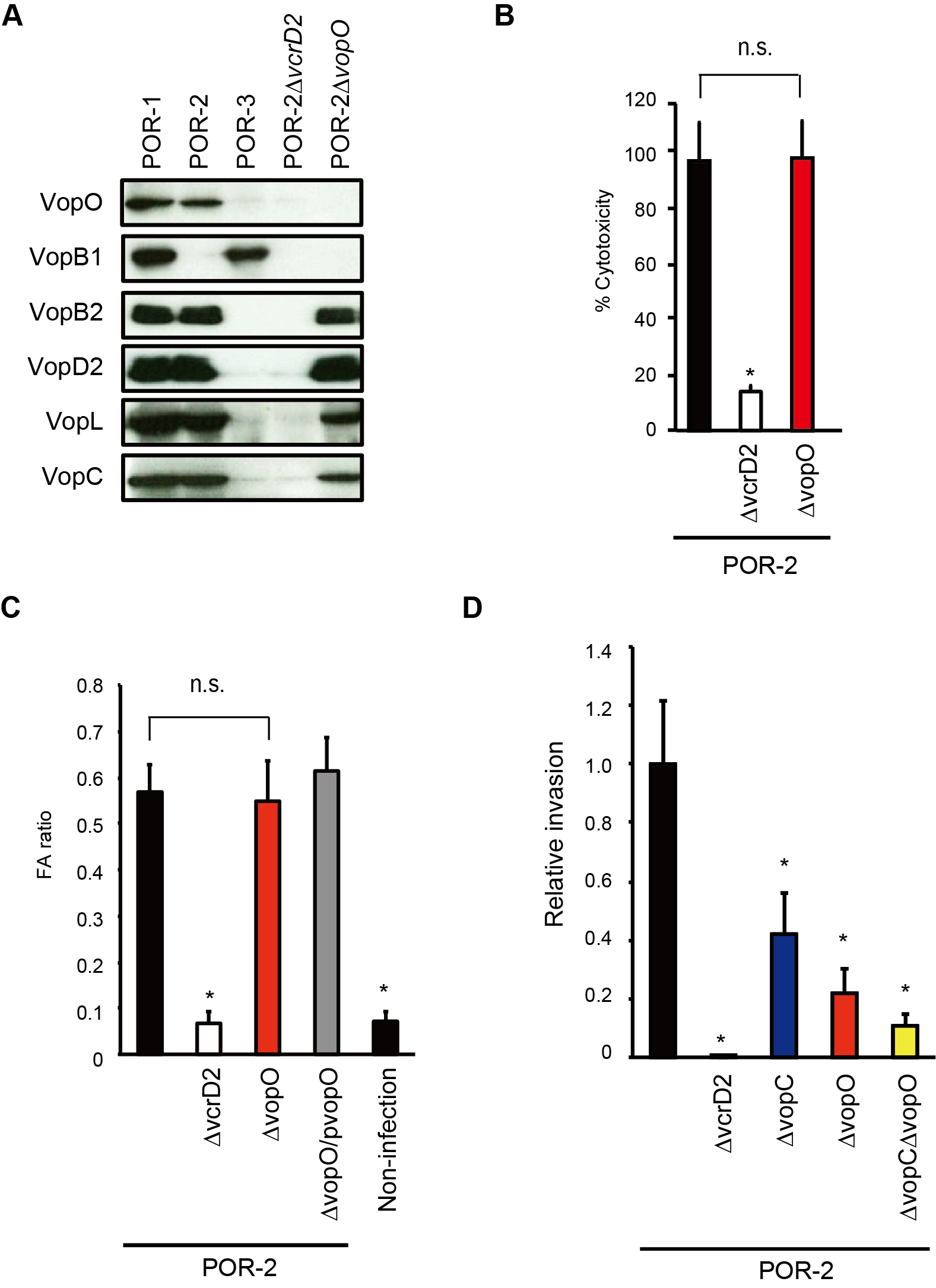

Supplement: S2 Fig — (A) Secreted protein profiles of POR-1 (tdhAS-deficient strain), POR-2 (T3SS1-deficient strain derived from POR-1), POR-3 (T3SS2-deficient strain derived from POR-1), POR-2∆vcrD2 (T3SS1- and T3SS2-deficient strain derived from POR-1), and POR-2∆vopO (vopO-deficient strain derived from POR-2). The membranes were probed with anti-VopO, anti-VopB1 (translocon of T3SS1), anti-VopB2 (translocon of T3SS2), anti-VopD2 (translocon of T3SS2), anti-VopL (T3SS2 effector), or anti-VopC (T3SS2 effector) antibodies. (B) Effect of vopO deletion on T3SS2-dependent cytotoxicity against Caco-2 cells. Caco-2 cells were infected with isogenic mutants of POR-2 at a MOI of 10. At 6 h after infection, cytotoxic activity was evaluated by determining the amount of lactate dehydrogenase released. The asterisks indicate results that differ significantly from those obtained using the wild-type strain (*p < 0.01). The error bars indicate the standard errors for experiments performed in triplicate. n.s. = not significant. (C) Effect of vopO deletion on T3SS2-dependent enterotoxicity. The enterotoxicity of various isogenic V. parahaemolyticus mutant strains was evaluated using the rabbit ileal loop test. The fluid accumulation (FA) ratio in each loop was measured 18 h after infection. FA is the amount of accumulated fluid (in ml) per length (in cm) of ligated rabbit small intestine. The asterisks indicate results that differ significantly from those obtained using the wild-type strain (*p < 0.01). The error bars represent the standard errors. n.s. = not significant. (D) Effect of vopO deletion on T3SS2-mediated cell invasion. HeLa cells were infected with isogenic mutant strains of POR-2 at an MOI of 2 for 2 h. Frequencies of invasion are expressed relative to the invasion of POR-2, which was set at 1.0. The error bars indicate the standard errors for experiments performed in triplicate. The asterisks indicate results that differ significantly from those obtained using the POR-2 strain (*p < 0.01 [file ppat.1004694.s002.tif]

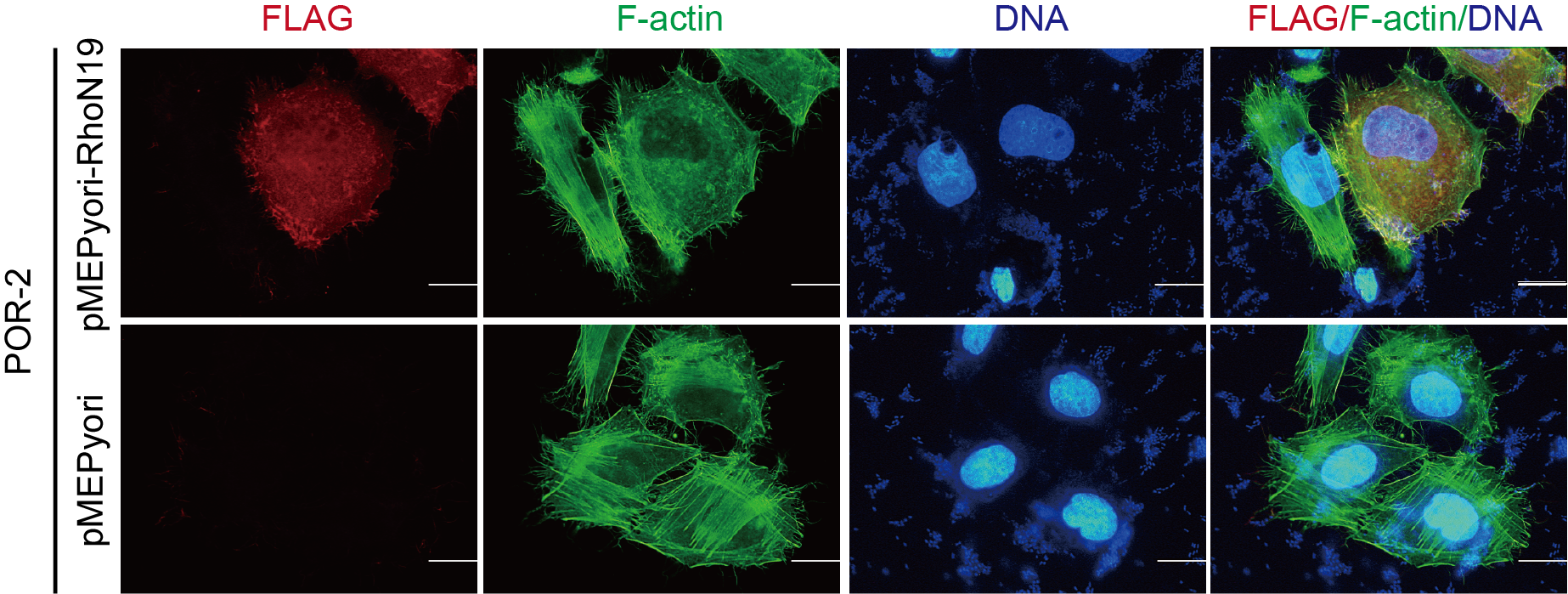

Supplement: S3 Fig — FLAG-tagged DN-RhoA (pMEPyori-RhoN19) or mock vector (pMEPyori) was transfected into HeLa cells. At 24 h after transfection, the cells were infected with a POR-2 strain at a MOI of 10 for 3 h. The cells were stained to detect transiently expressed DN-RhoA (red), F-actin (green), and cellular and bacterial DNA (blue). (TIF) [file ppat.1004694.s003.tif]

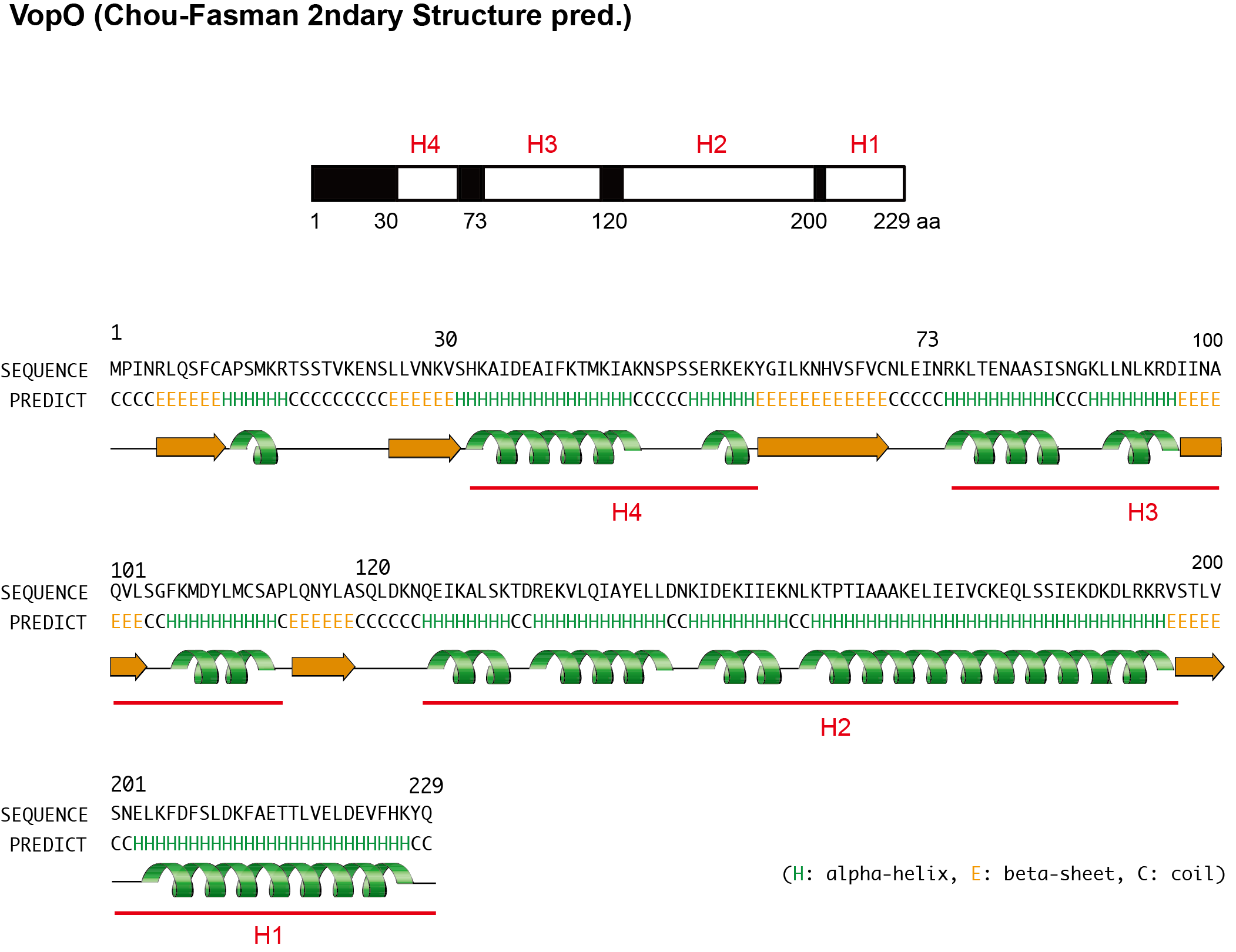

Supplement: S4 Fig — The secondary structure of the VopO protein was predicted using the Chou-Fasman secondary structure prediction program (http://cib.cf.ocha.ac.jp/bitool/MIX/). (TIF) [file ppat.1004694.s004.tif]

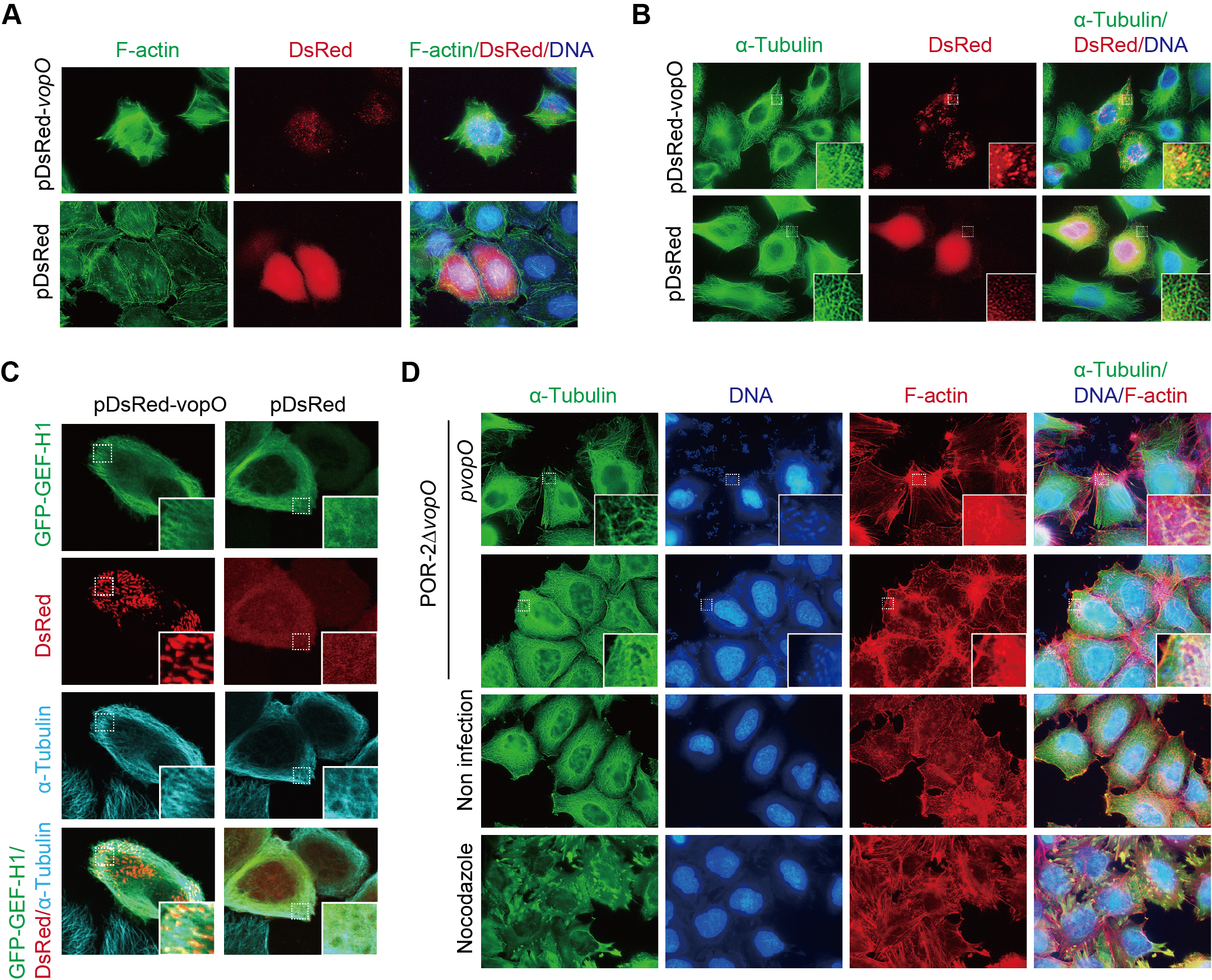

Supplement: S5 Fig — (A) Visualization of F-actin (green), DsRed (red), and cellular DNA (blue) in HeLa cells transfected with a VopO expression construct (DsRed-vopO) or empty DsRed vector (DsRed). (B) Effect of cellular VopO expression on the microtubule network. Cells transfected with a VopO-expression construct (DsRed-vopO) or empty DsRed vector (DsRed) were stained to detect tubulin (green) and cellular and bacterial DNA (blue). (C) Effect of cellular VopO expression on association of GEF-H1 with microtubules. A VopO expression construct (DsRed-vopO) or empty DsRed vector (DsRed) were co-transfected with GFP-GEF-H1 expressing vector (green). After staining to detect tubulin (cyan), the cells were examined by confocal microscopy. (D) Effect of V. parahaemolyticus infection on microtubule dynamics. HeLa cells were infected with a vopO mutant (POR-2∆vopO) and its complemented strain (POR-2∆vopO/pvopO) for 3 h. The cells were stained to detect tubulin (green), cellular and bacterial DNA (blue), and F-actin (red). (TIF) [file ppat.1004694.s005.tif]
