# Supplementary material for: Interaction between the Type III Effector VopO and GEF-H1 Activates the RhoA-ROCK Pathway
Source: PLoS Pathog. 2015 Mar 4;11(3):e1004694. doi: 10.1371/journal.ppat.1004694 (PMC4349864; doi:10.1371/journal.ppat.1004694)
Supplement: S1 Table — (DOCX) [file ppat.1004694.s006.docx]

**Table S1. Bacterial strains and plasmids used in this study**

| Strain or plasmid | Description | | Source or reference | |
| --- | --- | --- | --- | --- |
| ***V. parahaemolyticus*** |  |  | |  |
| WT (RIMD2210633) | Clinical isolate; KP positive, serotype O3: K6 | | [2] | |
| POR-1 | *tdhAS* null mutant strain: *tdhAS* deletion mutant derived from WT | | [52] | |
| POR-2 | TDH and T3SS1 deficient strain: *vcrD1* deletion mutant derived from POR-1 strain | | [52] | |
| POR-3 | TDH and T3SS2 deficient strain: *vcrD2* deletion mutant derived from POR-1 strain | | [52] | |
| POR-2*∆vcrD2* | TDH, T3SS1 and T3SS2 deficient strain: *vcrD2* deletion mutant derived from POR-2 | | [22] | |
| POR-2*∆vopO*  POR-2*∆vopC* | *vopO (vpa1329)* deletion mutant derived from POR-2  *vopC* deletion mutant derived from POR-2 | | This study  [7, 10] | |
| POR-2*∆vopL*  POR-2*∆vopC∆vopO* | *vopL* deletion mutant derived from POR-2  *vopC and vopO* deletion mutant derived from POR-2 | | [10]  This study | |
|  |  | |  | |
| ***E. coli*** |  | |  | |
| DH5α | F^-^ φ80*lacZ*ΔM15 *Δ(lacZYA-argF*)U169 *deoR recA1 endA1 hsdR17 phoA supE44 thi-1 gyrA96 relA1 λ*^-^ | | Laboratory Collection | |
| SM10 λ*pir* | *thi thr leu tonA lacY supE recA*::RP4-2Tc::Mu *λpir* R6K | | Laboratory Collection | |
| BL21 | F^-^ *dcm ompT hsdS*(r_B_^-^ m_B_^-^) gal [malB^+^]_K-12_(λ^S^) | | Laboratory Collection | |
|  |  | |  | |
| **Plasmids** |  | |  | |
| pCR2.1-TOPO | Multicopy (ColE1 *ori*) TA cloning vector, Amp^r^ | | Novagen | |
| pBlue-script II KS (-) | Cloning vector, Amp^r^ | | Stratagene | |
| pYAK1 | R6K-ori suicide vector for gene replacement, Cm^r^ | | [22] | |
| pYAK1-*ΔvopO* | Derivative of suicide vector pYAK1 for generating the *vopO (vpa1329)* deletion mutant | | This study | |
| pSA19CP-MCS | Complement vector for *V. parahaemolyticus*, Cm^r^ | | [22] | |
| *pvopO* | pSA19CP-MCS containing full length *vopO* gene | | This study | |
| *p∆H1* | pSA19CP-MCS containing *H1-*truncated *vopO* | | This study | |
| *p∆H12* | pSA19CP-MCS containing *H12-*truncated *vopO* | | This study | |
| *p∆H2* | pSA19CP-MCS containing *H2-*deleted *vopO* | | This study | |
| pEGFP-C1 | Expression vector of C-terminally GFP fused with inserted construct in host cell line. | | Clontech Laboratories | |
| pGFP-*vopO* | pEGFP-C1 containing of full length *vopO* gene | | This study | |
| pGFP-*∆H1* | pEGFP-C1 containing of *H1-*truncated *vopO* | | This study | |
| pGFP-*∆H12* | pEGFP-C1 containing of *H12-*truncated *vopO* | | This study | |
| pGFP-*∆H2*  pDsRed  pDsRed- *vopO* | pEGFP-C1 containing of *H2-*deleted *vopO*  Expression vector of C-terminally DsRed fused with inserted construct in host cell line  pDsRed-C1 containing of full length *vopO* gene | | This study  Clontech Laboratories  This study | |
| pGEX-6P-1 | Expression vector for GST tagged recombinant proteins in *E. coli*, Amp^r^ | | GE Healthcare Life Sciences | |
| pGEX-*vopO* | pGEX-6P-1 containing of full length *vopO* gene | | This study | |
| pGEX-*∆H1* | pGEX-6P-1 containing of *H1-*truncated *vopO* | | This study | |
| pGEX-*∆H12* | pGEX-6P-1 containing of *H12-*truncated *vopO* | | This study | |
| pGEX-*∆H2* | pGEX-6P-1 containing of *H2-*deleted *vopO* | | This study | |
| pCMV-TnT  pEGFP-GEF-H1 | Expression vector for *in vitro* transcription/translation system, Amp^r^  Expressing vector for GFP-fused GEF-H1 | | Promega  [47] | |
| pCMV-TnT -GEH-H1-3xFlag | pCMV-TnT containing of 3xFLAG tagged wt *gef-h1* gene | | This study | |
| pCMV-TnT-∆C-3xflag | pCMV-TnT containing of 3xFLAG tagged C terminal-truncated *gef-h1* gene | | This study | |
| pCMV-TnT-N-3xflag | pCMV-TnT containing of 3xFLAG tagged N terminal of *gef-h1* gene | | This study | |
| pCMV-TnT-DHPD-3xflag | pCMV-TnT containing of 3xFLAG tagged DHPH of *gef-h1* gene | | This study | |
| pCMV-TnT-∆N-3xflag | pCMV-TnT containing of 3xFLAG tagged N terminal-truncated *gef-h1* gene | | This study | |
| pCMV-TnT-C-3xflag | pCMV-TnT containing of 3xFLAG tagged C terminal of *gef-h1* gene | | This study | |
| pMEPyori 18Sf | Expression vector for eukaryotic cell | | [53] | |
| pMEPyori-RhoN19 | pMEPyori 18Sf containing dominant negative RhoA | | [54] | |
