# Supplementary material for: Interaction between the Type III Effector VopO and GEF-H1 Activates the RhoA-ROCK Pathway
Source: PLoS Pathog. 2015 Mar 4;11(3):e1004694. doi: 10.1371/journal.ppat.1004694 (PMC4349864; doi:10.1371/journal.ppat.1004694)
Supplement: S1 Text — (DOCX) [file ppat.1004694.s007.docx]

**Supporting Information**

**Supplemental Experimental Procedures**

**Cytotoxicity assays**

Cytotoxicity assays were performed as previously described [4]. Briefly, Caco-2 cells were infected at a MOI of 10. At 6 h after infection, the release of lactate dehydrogenase into the medium was quantified using a CytoTox96 kit (Promega, Fitchburg, WI, USA) according to the manufacturer’s instructions.

**Rabbit ileal loop test**

The rabbit ileal loop test was performed as previously described [4]. Isogenic mutant strains of *V. parahaemolyticus* (10^9^ CFU/loop) were injected into a ligated rabbit ileal loop, followed by measurements of fluid accumulation (FA) in each loop at 18 h after injection. The FA ratios were calculated as the amount of accumulated fluid (mL) per length (cm) of ligated rabbit small intestine. All of the animal experiments were performed according to an experimental protocol approved by the Ethics Review Committee for Animal Experimentation of the Research Institute for Microbial Diseases (Osaka University).

**Invasion assay**

The invasion assay was performed according to a previously published method [7]. Briefly, bacteria were grown in LB medium containing 0.04% crude bile for 3 h and then used to infect HeLa cells at an MOI of 2 for 2 h. After infection, the cells were incubated for 1 h in DMEM containing 10 µg/mL gentamicin for 1 h. The cells were then lysed in PBS containing 0.1% Triton X-100 and plated onto LB agar to determine the number of internalized bacteria. The invasion rate was calculated based on the mean number of colony-forming units of bacterial isolates internalized in cells relative to the number of bacteria in the original inoculum.

**Western blotting**

Proteins secreted by bacteria were prepared as described previously [4]. The samples used for western blotting were separated via SDS-PAGE. After electrotransfer, the PVDF membranes (Merck Millipore, Darmstadt, Germany) were probed with anti-VopB1, anti-VopB2, anti-VopD2, anti-VopC, anti-VopL, or anti-VopO (developed in-house) antibodies, and subsequently probed with horseradish peroxidase-conjugated goat anti-rabbit or rabbit anti-mouse antibodies (Zymed Laboratories, Inc., South San Francisco, CA, USA). The blots were developed using an ECL Western Blotting Kit (GE Healthcare, Little Chalfont, UK).

**Statistical analysis**

All of the data were expressed as the mean and standard error based on at least three determinations per experimental condition. Student’s *t* tests that assumed unequal variances were used for the statistical analyses. *P* < 0.05 was considered significant.

**Supplemental References**

52. Park KS, Ono T, Rokuda M, Jang MH, Okada K, et al. (2004) Functional characterization of two type III secretion systems of *Vibrio parahaemolyticus*. Infect Immun 72: 6659-6665.

53. Horiguchi Y, Inoue N, Masuda M, Kashimoto T, Katahira J, et al. (1997) *Bordetella bronchiseptica* dermonecrotizing toxin induces reorganization of actin stress fibers through deamidation of Gln-63 of the GTP-binding protein Rho. Proc Natl Acad Sci U S A 94: 11623-11626.

54. Akeda Y, Kodama T, Kashimoto T, Cantarelli V, Horiguchi Y, et al. (2002) Dominant-negative Rho, Rac, and Cdc42 facilitate the invasion process of *Vibrio parahaemolyticus* into Caco-2 cells. Infect Immun 70: 970-973.
